# The origins of unpredictability in life outcome prediction tasks

**DOI:** 10.1073/pnas.2322973121

**Published:** 2024-06-04

**Authors:** Ian Lundberg, Rachel Brown-Weinstock, Susan Clampet-Lundquist, Sarah Pachman, Timothy J. Nelson, Vicki Yang, Kathryn Edin, Matthew J. Salganik

**Affiliations:** ^a^Department of Information Science, Cornell University, Ithaca, NY 14853; ^b^Department of Sociology, Princeton University, Princeton, NJ 08544; ^c^Department of Sociology, St. Joseph’s University, Philadelphia, PA 19131; ^d^Office of Population Research, Princeton University, Princeton, NJ 08544; ^e^Center for Information Technology Policy, Princeton University, Princeton, NJ 08544

**Keywords:** life course, prediction, machine learning, mixed methods, limits to prediction

## Abstract

Why are some life outcomes difficult to predict? We investigated this question through in-depth qualitative interviews with 40 families sampled from a multidecade longitudinal study. Our sampling and interviewing process was informed by the earlier efforts of hundreds of researchers to predict life outcomes for participants in this study. The qualitative evidence we uncovered in these interviews combined with a mathematical decomposition of prediction error led us to create a conceptual framework. Our specific evidence and our more general framework suggest that unpredictability should be expected in many life outcome prediction tasks, even in the presence of complex algorithms and large datasets. Our work provides a foundation for future empirical and theoretical work on unpredictability in human lives.

## Significance

Scientists and decision-makers routinely make life outcome predictions: they use information from the past to predict what will happen to someone in the future. These predictions, whether made by human experts or algorithms, are often used to guide actions. Yet despite advances in artificial intelligence and predictive algorithms, life outcome predictions can be surprisingly inaccurate. We investigate the origins of this unpredictability through in-depth, qualitative interviews with 40 carefully selected families who are part of a multidecade research study. Their stories suggest origins of unpredictability that may apply broadly. Those who rely on predictions to inform high-stakes decisions about people should anticipate that life outcomes may be difficult to predict, even despite growing access to data and improved predictive algorithms.

Bella was born in a large American city around the year 2000.^*^ Bella’s family was not wealthy by any means, but both of her parents graduated from high school, they were married soon after she was born, and both had stable employment. Bella’s mom described Bella’s childhood this way: “She was nice and friendly, you know, just went to school and played and that was pretty much it.” But by the time Bella turned 15, things looked very different. She was getting in fights at school and struggling in class. Eventually, she dropped out of high school. Could Bella’s transition from a happy childhood to struggling adolescence have been predicted?

Questions like this about the predictability of human outcomes have been the subject of research and speculation at least since Cicero published *On Divination* in 44 BCE (1). Although these questions usually seem intractable, in Bella’s case we can be unusually confident in our answer. Bella was part of a multidecade longitudinal social science study that collected detailed information about the life outcomes of thousands of families (2). Then, hundreds of researchers used these data to create algorithms that predicted grades at age 15 and five other specific life outcomes (3). Of all the algorithms trained on this rich dataset, the very best algorithm was not very accurate for Bella or overall.

The goal of predicting someone’s future might seem more rooted in science fiction than science, but life outcome predictions are actually quite common: doctors predict the outcomes of patients, social workers predict the risk of mistreatment of children, landlords predict whether potential tenants will pay their rent, firms predict the productivity of potential employees, banks predict the creditworthiness of potential borrowers, and judges predict the likelihood that someone who was arrested will appear at trial.

While humans have historically carried out life outcome predictions unaided, there is increasing interest in making life outcome predictions using complex algorithms trained on large datasets. For example, Kleinberg et al. (4) estimate that replacing judges with a machine learning model for decisions about bail could result in 40% fewer people being subjected to pretrial detention with no increase in crime and with a decrease in racial disparities. There are appropriate concerns about the fairness, accountability, transparency, ethics, and utility of these algorithms (5–8), as well as cautious optimism that carefully designed algorithms might improve decisions and, by extension, well-being (4, 9).

Despite the fact that life outcome predictions are common and often involved in high-stakes decisions, they have not been the focus of research about the life course (for a few exceptions, see ref. 10). This limited attention means that there is little scientific foundation for understanding the degree of accuracy in these predictions. Nor is there an understanding of the fundamental processes that determine the predictability of life outcomes, and whether these might be overcome with more data, better algorithms, and improved theory.

In order to understand the origins of unpredictability in one particular outcome—grades at age 15—we spoke with 40 families within a multidecade longitudinal study. Our sampling and interviewing processes were informed by the earlier efforts of hundreds of researchers to predict life outcomes for participants in this study. These interviews led to a conceptual framework that helps us understand unpredictability.

## 1. Conceptual Framework

**Fig. 1.**
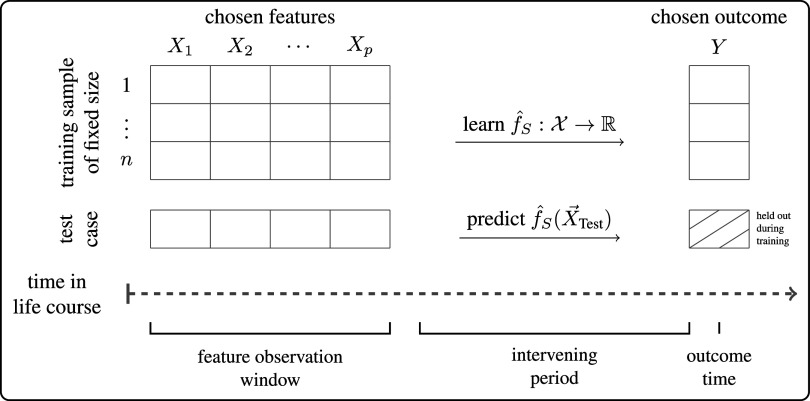
Life outcome prediction task. In the task we studied, the feature observation window is from the child’s birth to age 9. The intervening period follows from age 9 to 15. The outcome time is age 15. The chosen features are 12,942 survey responses. The chosen outcome is self-reported grade point average (GPA), which ranges from 1.00 (worst) to 4.00 (best).

We define a life outcome prediction task by three elements (Fig. 1): 1) A set of features (predictor variables) measured about a person and their environment. We refer to the time when features are measured as the feature observation window. In the task we studied, the features are a specific set of childhood experiences measured from birth to age 9. 2) An outcome variable, which we require to be measured at some point after the feature observation window. In the task we studied, the outcome is GPA at age 15. Between the feature observation window and outcome measurement is an intervening period, such as the 6 y between age 9 and 15. We refer to the length of the intervening period as the time horizon. 3) The process that produces a training sample; this element includes both the sampling method and the sample size. An example is a simple random sample of a given size from a particular population.

Once a life outcome prediction task is defined, people attempt the task using a learning approach: any procedure to create an algorithm that takes as inputs feature values and returns a predicted outcome. We conceptualize the learning approach broadly to include any combination of human decision-making and machine learning.

In this paper, we measure performance by mean squared out-of-sample prediction error. Mathematically, let $\left(\vec{X},Y\right)$ denote the feature vector and outcome for a random unit from the population, and let ${\hat{f}}_{S}$ denote a prediction function learned in the training sample S. Out-of-sample mean squared error is an estimate of expected squared error $\;\text{E}({\left({\hat{f}}_{S}\left(\vec{X}\right)-Y\right)}^{2})$ with expectation taken over randomness in $\vec{X}$, Y, and S.

The key step in our framework is to transform each life outcome prediction task into a mathematically equivalent estimation task. This transformation enables us to naturally decompose prediction error into two mutually exclusive, exhaustive, and interpretable components.

Suppose there was only one binary feature X. Suppose we knew the subpopulation mean outcomes E(Y∣X=0) and E(Y∣X=1) among those with feature value X=0 and X=1, respectively. When presented with a new case with feature value X=1, the prediction that would minimize expected squared error would be the mean outcome in that subgroup, E(Y∣X=1). The task of predicting this outcome is equivalent to the task of estimating this mean. To generalize this notion beyond the univariate case, define a set of people as *observationally identical* if they share the same values on all chosen features $\vec{X}$. The task of predicting Y given a feature vector value $\vec{X}=\vec{x}$ is the same as the task of estimating the mean outcome $\;\text{E}(Y|\vec{X}=\vec{x})$ within the set of observationally identical people whose features take the vector value $\vec{x}$.

**Fig. 2.**
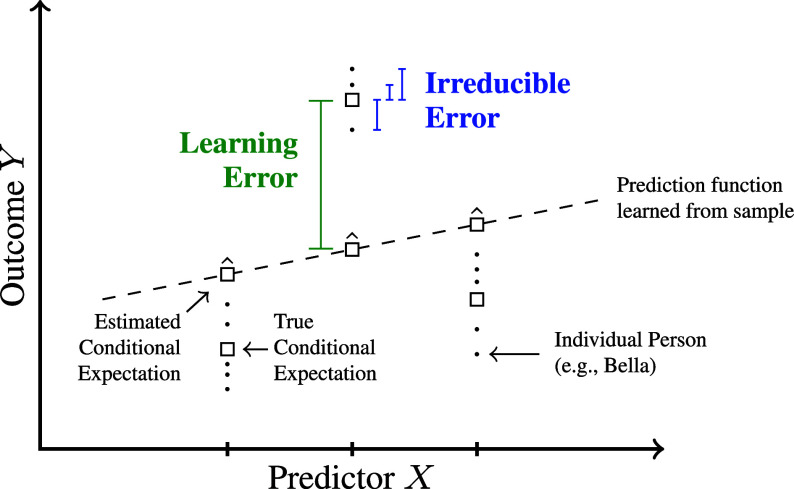
Origins of unpredictability: irreducible error and learning error. See also figure 1.6 in ref. 12. Each dot represents a person; people who share a single value on the predictor have many outcomes (each vertical set of dots). This type of error is fixed by the task definition, so we call it *irreducible error*. Second, the estimated prediction function (regression line) is not equal to the true conditional expectation in each subgroup defined by X. The prediction is wrong in this case because the true relationship is not linear, but it could also be wrong due to sampling variability or bias. Because this component relates to the learning procedure, we call it *learning error*.

The estimation perspective partitions two origins of error when producing a prediction for the outcome of an individual person (Fig. 2). First, the person’s outcome may be far from the average outcome among all observationally identical people. Second, the predicted outcome may be far from the subgroup average outcome. These two origins are conceptually distinct: The first involves the individual’s outcome but does not involve the predicted value, and the second involves the predicted value but does not involve the individual’s outcome.

The two origins of error can be averaged over persons to yield a mathematical decomposition of expected squared error.

[1] $$\begin{array}{cc} & \underset{\begin{array}{c} Prediction\;Error\\ \;\text{Expected squared}\\ \;\text{prediction error} \end{array}}{\underbrace{\;\text{E}\left({\left[Y-{\hat{f}}_{S}\left(\vec{X}\right)\right]}^{2}\right)}}\\ & \;=\underset{\begin{array}{c} Irreducible\;Error\\ \;\text{Outcome variance}\\ \;\text{given predictors} \end{array}}{\underbrace{\;\text{E}\left(\;\text{V}\left[Y|\vec{X}\right]\right)}}+\underset{\begin{array}{c} Learning\;Error\\ \;\text{Expected squared error}\\ \;\text{for the conditional mean} \end{array}}{\underbrace{\;\text{E}\left({\left[{\hat{f}}_{S}\left(\vec{X}\right)-\;\text{E}\left(Y|\vec{X}\right)\right]}^{2}\right)}}. \end{array}$$

The term we have labeled *irreducible error* is the average squared difference between each person’s outcome and the true (but unknown) mean among people who are observationally identical to them, which is the within-group variance. We call it irreducible error because within-group variance is fixed by the features and outcome and does not involve the predicted values. Irreducible error cannot be reduced by a new learning procedure; the only way it can be decreased is by changing the task. The term we have labeled *learning error* is the average squared difference between the estimated and true within-group mean outcomes. We refer to this component as learning error because it corresponds to errors in the learned prediction. Irreducible error and learning error additively comprise expected squared error (Eq. **1** and *SI Appendix*, section 4) (11).

**Fig. 3.**
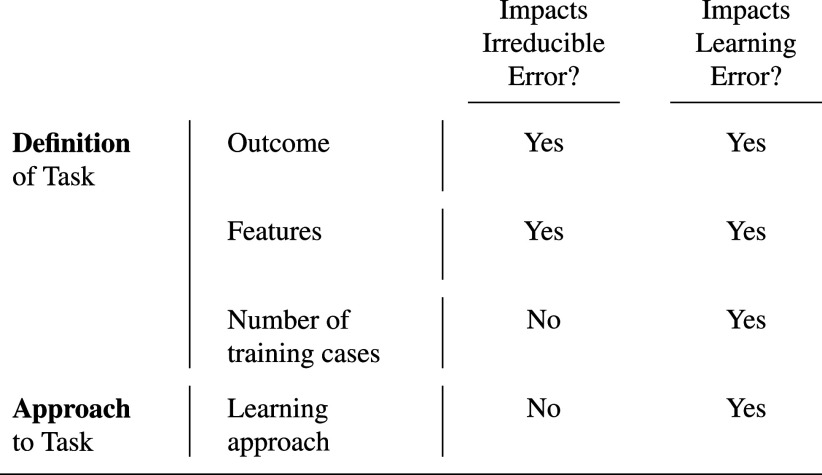
Inputs to irreducible error and learning error.

Some researchers further decompose learning error into model approximation and estimation error (12). Others further decompose learning error into bias and variance (11). We focus on irreducible and learning error because these two components have conceptually distinct sources: irreducible error is a function of the task only, whereas learning error is a function of both the task and the learning approach (Fig. 3). We conceptualize the learning approach to include all decisions made by the researchers when going from the raw data to the final predictions. Our conceptual framework applies to any prediction function ${\hat{f}}_{S}$ no matter how it is created: any kind of statistical learning, human expertise, or combination of the two.

## 2. Data

By taking a mixed-methods approach (13, 14), we arrived at a framework that conceptualized unpredictability as originating in irreducible and learning error. Our team of 16 researchers conducted 114 semistructured, qualitative interviews with 73 respondents in 40 families.

Every family we interviewed was part of the Future of Families and Child Wellbeing Study (FFCWS; formerly the Fragile Families and Child Wellbeing Study), a multidecade longitudinal study tracking the lives of thousands of families who gave birth around the year 2000 in 20 large U.S. cities (2). Researchers collected survey data in five waves from the birth of the child through age 9, and then again in a sixth wave when children were 15 y old. The study gathered data from many respondents (child, child’s parents, primary caregiver, teacher, etc.) on many different topics (material resources such as income, social factors such as parents’ relationship, school characteristics, perceptions of the residential neighborhood, etc., *SI Appendix*, Fig. S3). Data also include psychometric testing of the child’s cognitive development. Because of their depth, breadth, and quality, the FFCWS data have been used in more than 1,000 published papers (15).

To select a sample of families from FFCWS that would be especially informative about the origins of unpredictability, we drew on the results of the Fragile Families Challenge (3). The Challenge was a scientific mass collaboration in which hundreds of researchers used the FFCWS data for six life outcome prediction tasks. We focus on one of these tasks, in which researchers predicted each child’s average self-reported grades in four subjects: English, history, math, and science. We refer to this outcome as grade point average (GPA), which can range from 1.00 (worst) to 4.00 (best). The 12,942 features were collected in the FFCWS from the birth of the child through age 9, including features such as family income, parental relationship status, and teacher reports of child behavior and school performance. The training set was 2,121 cases for which participants had access to the GPA at age 15. The task thus involved predictions over a six-year time horizon, from age 9 to age 15. Performance was evaluated on a holdout set by $R_{\text{Holdout}}^{2}$, which rescales out-of-sample mean squared error so that a score of zero corresponds to predicting the mean of the training data and a score of one corresponds to perfect prediction. Despite using a rich dataset, a variety of theoretical approaches, and state-of-the-art machine learning, no researchers were able to make very accurate predictions: the best $R_{2}^{\text{Holdout}}$ when predicting GPA was 0.19 (3, 16). Predictability was also low for the other five prediction tasks with other outcomes.

The most accurate algorithm from the Challenge (17) provides a useful approximation for the best possible predictions for this task, given the expertise—substantive and methodological—available at that time. As such, children’s outcomes that are not well-predicted by this algorithm may be particularly informative about the origins of unpredictability in this task. Therefore, for our study, we oversampled children whose GPAs were much higher than predicted and much lower than predicted. To avoid concerns about overfitting, we limited the sampling frame to children who were not in the training set. To capture the full distribution of predicted values, we stratified the sampling frame into terciles based on predicted GPA and conducted our sampling within terciles. To reduce the risk of misinterpreting the experiences of outliers, we also sampled some children whose actual and predicted GPA were similar. To increase our chance of observing structural forces that might be invisible to participants, we sampled children born in three different cities. Finally, to reduce the risk of motivated measurement, the primary interviewer for each case was not told the predicted and realized GPA. Our sample size of 40 families was determined by budget constraints. For additional information about the sampling and interview procedure, see *Materials and Methods* and *SI Appendix*, sections 2 and 3. This design—which combines ideas from the qualitative and quantitative research traditions—was created to be informative about the origins of unpredictability.

## 3. Results: Origins of Unpredictability

### 3.1. Sources of Irreducible Error.

**Fig. 4.**
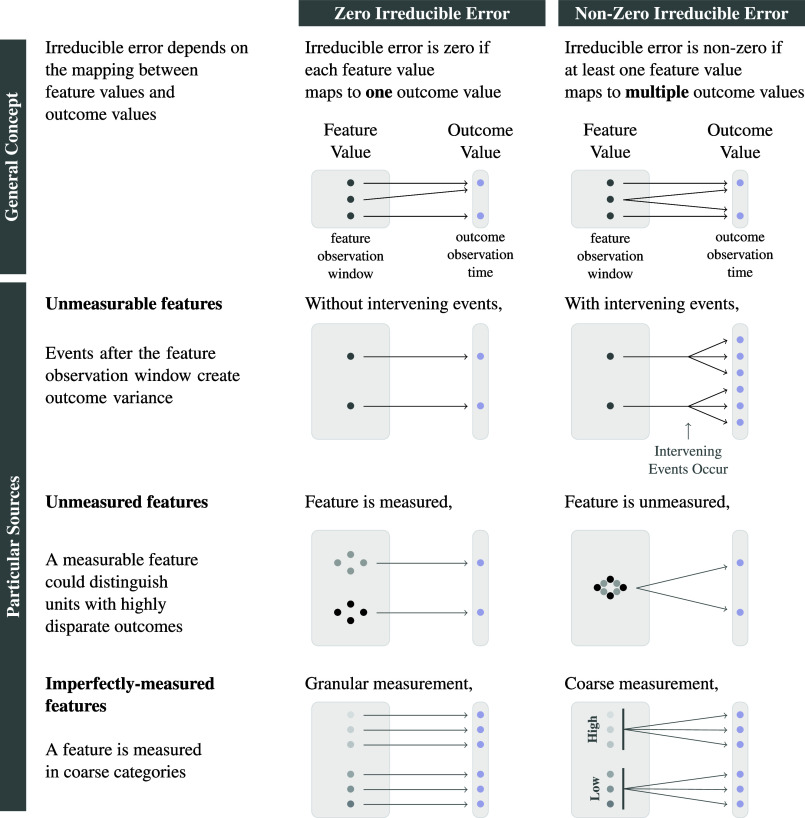
Sources of irreducible error.

Irreducible error exists to the degree that observationally identical children have outcomes that vary (Fig. 2). From our interviews, we identified three nonexhaustive sources of irreducible error that organize what we found and that may apply to other life outcome prediction tasks. These three sources are: 1) *unmeasurable features* that occur after the feature observation window, 2) *unmeasured features* that could have been measured because they occur during the feature observation window, and 3) *imperfectly measured features* (Fig. 4).

#### 3.1.1. Unmeasurable features: Consequential intervening events.

In a life outcome prediction, time elapses between measurement of predictors and realization of an outcome. Events in the intervening period, which we call consequential intervening events, cannot be measured and can create irreducible error. Our interviews revealed some consequential intervening events that upended children’s lives. This was true for Bella, the youth whose story opened the paper. Bella’s grades may have been unexpectedly low because of events which occurred after all predictors were measured: Her father died unexpectedly, her mother became depressed, and as Bella found herself effectively parentless she began to struggle academically.

The interviews also revealed consequential intervening events that were more fleeting, but which were consequential for grades nonetheless. Charles attended an online charter school and mostly performed well, working in the family dining room upstairs under parental supervision. But in the specific term for which GPA was measured, he attended school from the basement, where he often played online video games. That semester, Charles reported a 1.75 GPA, much lower than the predicted value of 3.15. Subsequently, his mother became concerned and said “no more downstairs.” His grades recovered: “Ninth grade, I did terrible, then all the other years, I did As.”

For both Charles and Bella, an event occurred after all predictors were measured: Bella’s father died, and Charles briefly attended school from his basement. These events may have caused their outcomes to differ from others who were observationally identical. Yet the relevant events could not have been measured: they occurred during the period between predictors and outcomes. Consequential intervening events are an important source of irreducible error, particularly for life outcome prediction tasks with long time horizons like that from age 9 to age 15.

#### 3.1.2. Unmeasured features.

Some features could have been measured during the feature observation window, yet they were unmeasured. Features may be unmeasured for good reason, such as a survey designer facing a budget constraint. Yet unmeasured features can create irreducible error to the degree that they are independent of the measured predictors and relevant to the outcomes of many cases.

Our qualitative interviews did not reveal a small set of additional predictors that we think would have greatly improved predictive performance. This is perhaps unsurprising—the FFCWS data included thousands of predictors collected with guidance from sociologists, psychologists, economists, and social work scholars. Yet, we did find examples of unmeasured predictors that seemed to be important in specific cases.

For example, Lola’s social network was particularly important when she was young, a period when her mother was dabbling in illegal activities that put her family at risk. Lola was able to consistently attend school during this time because an elderly neighbor got her ready each morning. Lola’s grandparents provided health insurance and an address to enroll her in a better school, and ultimately remodeled their basement so that Lola and her mother could move in. In recent years, her mother was stably employed by an aunt in a family business. Perhaps if Lola’s network had been measured, an algorithm could have better anticipated her 3.75 GPA, which outpaced the predicted value of 3.04.

#### 3.1.3. Imperfectly measured features.

Sometimes a feature was measured during the feature observation window, but it was measured imperfectly. This imperfect measurement can create irreducible error. When considering imperfect measurements, researchers often focus on respondent misreporting (18), but it can come from many other sources as well. For example, imperfect measurement can also arise from limitations inherent to survey research when a continuous construct may be measured in coarsened categories.

For example, respondents at age 9 answered a question “How close do you feel to your mom?” with four response options from “extremely close” to “not very close.” Hennessey chose not very close. Her actual GPA of 1.25 was far below the predicted value of 2.71. One explanation for this poor prediction is coarse measurement: She needed an answer choice beyond not very close. In our qualitative interview, Hennessey reported that at times when she needed her mother, her mother “blatantly ignored me.” The two bickered and physically fought. Her mother sometimes kicked her out of the house or called the police. When asked directly if her stressful home life impacted her school performance, Hennessey noted that it “affected me a lot.” She recalled a particular incident when her mother told her that “[y]ou better start treating me better, because I might not live that long.” This warning was so frightening that she went to the principal’s office because “I couldn’t even focus in class...I was shaking. That was all I could think about. I was, like, crying in school, and they [school staff] had no idea what was wrong with me.” Ultimately, Hennessey failed 8th grade and reported a low GPA in the FFCWS survey at age 15.

Hennessey’s turbulent relationship with her mother was very consequential in her life, and it was only coarsely captured by the survey data. To a trained model, she appears the same as any other respondent whose relationship with their mother was “not very close,” even though hers was likely much worse than theirs. Imperfect feature measurement thus made it harder to predict Hennessey’s outcome.

### 3.2. Sources of Learning Error.

Learning error exists to the degree that predicted values ${\hat{f}}_{S}\left(\vec{x}\right)$ are far from the conditional mean $\;\text{E}(Y|\vec{X}=\vec{x})$ (Fig. 2) in the population. Here, we focus on what makes learning error high in life outcome prediction tasks, especially with survey data: these tasks are likely to involve many features with a limited number of cases and limited amounts of expert knowledge. These characteristics together make conditional means difficult to estimate.

Life outcomes are the consequence of many inputs. For this reason, tasks are likely to involve many features. Our case study task involved 12,942 features selected by domain experts for their relevance to the life course. Even if each feature were binary, the number of possible feature vectors would be $2^{12,942}$, substantially more than the number of people who have ever lived (19). The impossibility of learning in such a space might suggest that tasks should be defined with fewer features. But concerns about irreducible error point the opposite direction: Our qualitative interviews suggest that accurate prediction might actually require even more features. For example, one child told us about a wealthy out-of-state family who mentored him since they were connected through a program for urban youth when he was in middle school. Another told us about a landlord who took an interest in his family (the tenants) and voluntarily built a home gym in the basement so the youth could follow his passion for fitness. If we wanted irreducible error to be closer to zero, we might choose life outcome prediction tasks with an even bigger number of features.

When there are many features, however, learning is possible only with a vast number of cases and/or a vast amount of expert knowledge outside of the data. The number of cases is limited by practical constraints; the costs of following people over time imply that longitudinal surveys typically involve only a few thousand people at most. In the absence of a vast number of cases, one could lean on expert knowledge. Perhaps an expert could somehow specify a small number of features—either in the original data or derived from the original data—that allow for accurate predictions? We see no evidence that such expert knowledge currently exists about the life course, and it is unclear whether it will ever exist.

Machine learning may seem to offer a way out: Perhaps an expert could narrow the class of possible models so that the data could then choose the best among the candidates. Suppose an expert narrows the feature space from 12,942 to 1,000 features, and argues for a linear model with interactions involving no more than two variables at a time. But then there are 1,000 main effects and (1,000 choose 2) interaction terms for machine learning to choose among: a total of 500,500 parameters. It is unreasonable to expect machine learning to magically find that many of these parameters are truly zero and also estimate the nonzero ones precisely. Machine learning is certainly a step forward; it might yield a sparse approximation that is better than what an expert would produce alone. But the magnitude of learning error may still be high in an absolute sense.

## 4. Discussion

This paper defined life outcome prediction tasks and used qualitative interviews to study unpredictability in one task. We identified a connection between our specific qualitative observations and a general decomposition of prediction error. We now reach beyond our specific evidence to argue that predictability will generally be low for many life outcome prediction tasks because of irreducible error and learning error. We conclude by discussing implications for policy and for science.

### 4.1. Generalizing to Other Life Outcome Prediction Tasks.

Because we study only one task, we cannot draw firm conclusions about predictability in general. We cannot say, for example, whether an outcome other than grades might be more or less predictable with the cases and features in our study, or whether a coarsened version of grades (e.g., failing vs. not) might be more or less predictable.

**Fig. 5.**
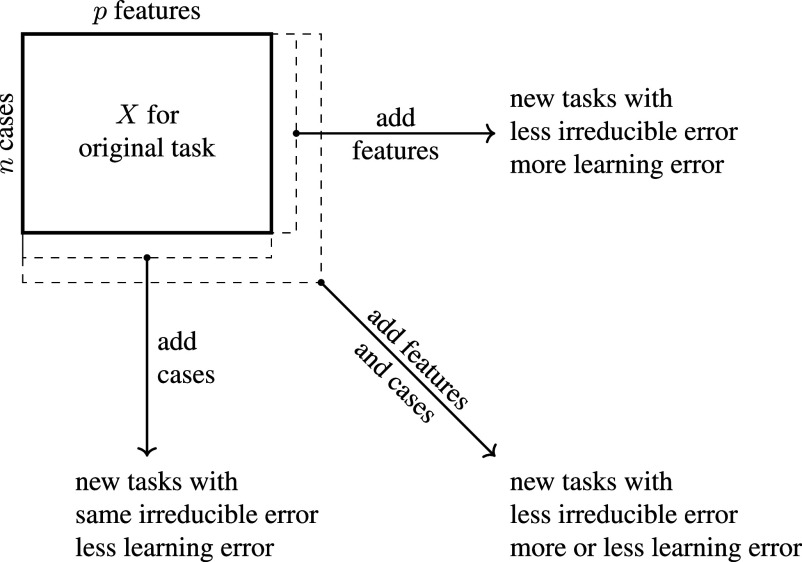
Generalizing to other life outcome prediction tasks.

For any particular outcome, researchers may believe that high prediction error has an easy answer: more data. It is hard to evaluate this claim because more data could mean three different things—more cases, more predictors, or both—each with different implications for irreducible and learning error (Fig. 5).

With more cases, learning error would be smaller but irreducible error would be unchanged. With more features, irreducible error would be smaller but learning error might increase because the number of unique feature values would grow exponentially with the number of features [the curse of dimensionality, (11)]. With more cases and more features, prediction error might decrease or increase: irreducible error might decrease, but learning error might increase to the degree that the number of added cases is insufficient to learn accurately about the added features.

In practice, cost constraints limit the number of features and cases when life outcome predictions are made with longitudinal survey data. Digital and administrative data held by companies and governments may offer qualitatively more cases and features (20). However, the features measured in digital and administrative data may be less useful for prediction than those measured in surveys (21, 22). Ultimately, predictability with administrative data remains an empirical question.

Yet our conceptual framework leads us to speculate that for life outcome prediction tasks using longitudinal survey data, low levels of predictability will be the norm. The sources of irreducible and learning error in our setting are likely to exist in many life outcome prediction tasks. Two classes of tasks that might deviate from this pattern are: 1) tasks for which a natural low-dimensional representation maps predictors to outcomes, such as when a lagged outcome is a good predictor of that outcome in the future and 2) tasks for which the time horizon is very short (e.g., 1 d). However, we think that natural low-dimensional representations and short time horizons are the exception rather than the norm for life outcome prediction tasks of interest in policy and science. Ultimately, our speculation requires empirical verification, refutation, and refinement. The strongest evidence will come from predictions preregistered before the outcomes have taken place (e.g., ref. 23) or from projects using the common task method (e.g., ref. 3).

### 4.2. Implications for Policy and for Science.

Much of the excitement about prediction for policy may stem from a belief that big data and machine learning magically lead to accurate predictions. While this may be true in some domains, we show that for life outcome prediction tasks, there are deep reasons to expect unpredictability. Therefore, decision makers should reorient their expectations and anticipate that life outcome predictions—generated by humans or by algorithms—may be inaccurate. Further, decision makers should recognize that in many practical situations accurate prediction is a means to an end, not an end in itself (24). In these cases, decision makers should focus less on accuracy and more on impact: the extent to which decisions informed by improved prediction actually lead to better outcomes (8, 25–28).

Individual-level prediction has not historically been a goal for many sociologists, yet the role of prediction in sociology (29–32) and the social sciences (33–37) has been the subject of growing debate in recent years. Our study does not argue for or against the view that researchers should strive to produce accurate predictions. Rather, we treat predictive accuracy as an empirical pattern to be understood, just like other patterns. Should others share this view, one way to build this understanding is to interview at least some cases following our design. Another way is to quantify patterns in both means and variances. Patterns in mean outcomes between groups have been the focus of much past research in sociology (e.g., difference in average life expectancy for people in different demographic groups) (38). Limits to predictability pose no direct threat to a focus on variation in mean outcomes across groups; group means can be well-estimated even if the outcomes of individuals within each group vary substantially (39, 40). However, limits to prediction suggest an important complementary goal: describing within-group variability (41). Just as researchers currently seek to identify the causes of between-groups differences, one can imagine a parallel search for the causes of within-group differences (42). A pivot from focusing on between-group variability to focusing on within-group variability could be a bridge between existing social science research traditions and research focused on limits to life outcome prediction.

Finally, researchers who want to focus specifically on the limits of predictions for life outcomes could estimate prediction error for many tasks where one aspect of the task is systematically varied (e.g., the feature sets, outcomes, sample sizes) (22, 43). These empirical studies would be particularly valuable if they could estimate not just prediction error, but also irreducible error and learning error, which is possible in at least some settings (44). Such empirical studies might support modeling efforts that help reveal the social processes that lead to irreducible and learning error (10) and models that yield sharp predictability bounds for specific data generating processes (45). This work on fundamental limits to life outcome prediction may be informed by research about fundamental limits to prediction in other fields, such as meteorology (46, 47) and financial markets (48). Ultimately, this research would lead to better frameworks for understanding life trajectories like Bella’s.

## Materials and Methods

Our interviews were designed to study unpredictability. Each interview traced the life history of the young adult from birth through the time of the interview. The interview guide focused on three periods: 1) the feature observation window of birth to age 9, 2) the intervening period of age 9 to 15, and 3) age 15 to the time of the interview, which was after outcomes were measured. *SI Appendix*, sections 6 and 7 provide the interview guides. Interviews were conducted in pairs, where only one of the two interviewers was aware of the outcome while conducting the interview. The interviewer who did not know the outcome conducted the interview. The interviewer who was aware of the outcome asked follow-up questions at the end. All interviews were recorded and transcribed. *SI Appendix*, section 3 describes the interview procedure. Data collection was approved by the Princeton University IRB (#10564), with informed consent obtained from all participants.

We analyzed each interview inductively. Several members of the team independently answered a series of questions about each case and then met to discuss it. The themes in the paper emerged from these discussions. As themes crystallized, we switched to a format where a single researcher would write a case summary, other researchers would read the case summary and interview, and then we would meet to discuss and finalize the summary.

## Supplementary Material

Appendix 01 (PDF)

## Data Availability

Some study data available. (Quantitative data are currently available to approved researchers from the Future of Families and Child Wellbeing Study: https://ffcws.princeton.edu/ (49). Researchers who are interested in analyzing the redacted transcripts from this study should contact the authors for more information.)

## Footnotes

^*^All names in this paper are pseudonyms chosen by the respondents.
