# Supplementary material for: The origins of unpredictability in life outcome prediction tasks
Source: Proc Natl Acad Sci U S A. 2024 Jun 4;121(24):e2322973121. doi: 10.1073/pnas.2322973121 (PMC11181083; doi:10.1073/pnas.2322973121)
Supplement: Supplementary file 1 — Appendix 01 (PDF) [file pnas.2322973121.sapp.pdf]

Supporting Information for

The origins of unpredictability in life trajectory prediction tasks

I. Lundberg, R. Brown-Weinstock, S. Clampet-Lundquist, S. Pachman, T.J. Nelson, V. Yang, K. Edin, M.J. Salganik

Correspondence: Kathryn Edin
E-mail: kedin@princeton.edu

This PDF file includes:

Supporting text
Figs. S1 to S3
Table S1
SI References

Supporting Information Text

1. Acknowledgments

We thank the following people who served as interviewers in this project: Bobbi Brashear, Rachel Brown-Weinstock, Maria Canals, Kristin Catena, Susan Clampet-Lundquist, Katie Donnelly, Kathryn Edin, Kaitlin Edin-Nelson, Alexus Fraser, Sarah Pachman, Ashley Hyman, Ian Lundberg, Stefanie Mavronis, Timothy Nelson, Matthew Salganik, and Vicki Yang.

We thank the following people for feedback on the manuscript: Abdullah Almaatouq, Alyssa Battistoni, Jennie E. Brand, Elizabeth Bruch, Emily Cantrell, Kyla Chasalow, Siwei Cheng, Diag Davenport, Ben Edelman, Sayash Kapoor, Jennifer Lee, Jakob Mokander, Jonathan Morduch, Daniel Rigobon, Brandon Stewart, Beza Taddess, Keyon Vafa, Akshay Venkatesh, and Haowen Zheng.

2. Detailed Sample Information

This project is embedded within the Future of Families and Child Wellbeing Study (FFCWS), a probability sample of children born in 1998–2000 in U.S. cities with populations over 200,000. The study is clustered in cities of birth. The Fragile Families Challenge assessed predictability of life outcomes in a subsample of 4,242 children born in 18 U.S. cities. As described by (1), half of the sample (2,121 families) were provided to social and data scientists to build predictive models, one-eighth of the sample (530 families) were used to provide instant feedback on the predictive performance of submissions, and three-eights of the sample (1,591 families) were held out and used to evaluate predictive performance at the end of the Challenge.

A. Sample Selection Process: Selecting Families for Qualitative Interviews. The sample for qualitative interviews was drawn from among the 1,591 families in the holdout set. We focused on the children born in three FFCWS cities who were not missing the outcome variable (Grade Point Average, hereafter GPA) at age 15. Our sampling strategy sought to (1) assign non-zero sampling probability to every family, (2) ensure representation across cities and predicted GPA, and (3) ensure representation across residual GPA, with oversamples of those with GPAs much higher and much lower than expected.

Fig. S1 illustrates the resulting sampling strategy for one city. We stratified families into terciles within each city based on the GPA that was predicted by the most accurate submission to the Challenge. This produced three equally-sized strata: those with low, middle, and high predicted GPAs. Next, we stratified within each (city $\times$ predicted GPA) stratum by the residuals of the prediction: how much the actual GPA was better or worse than predicted. We sampled the families with the most positive and negative residuals with probability 1. We hoped these families would be especially informative for learning about unpredictability. We then partitioned the remaining observations into terciles of residual GPA: low, middle, and high. In each of these terciles, we randomly sampled 1 out of the 6-9 families.

Overall, this design yielded a full sample of 45 families with 15 per city (Fig. S1). The 15 include 5 each whose predicted outcomes are low, medium, and high. Each set of 5 within a stratum of predicted GPA includes the two respondents with the most unexpected outcomes and three respondents whose outcomes capture a range of the remaining residual values.

Our choice to sample 45 families was driven by limitations of budget and time. We consider the appropriate sample size for this type of analysis to be an open question, for which the answer is likely to depend on the setting. In some settings, one might analyze data continuously as data are collected and continue gathering data as long as new information is being discovered. Our design, in contrast, sampled all families before data were collected; we did not change the sample size as a function of the results, and we carried out most of the analysis after all data were collected. There are important open questions about how one might carry out a similar analysis in settings with data-adaptive sampling or in settings with orders of magnitude more cases, which might require a different analytic approach.

B. Respondent Recruitment Process: Nonresponse, Refusals, and Replacements. Fig. S2 shows the final set of responding families. We succeeded in speaking with at least one of the youth or primary caregiver in 40 families, of whom 38 were chosen as part of the initial sample selection process. The responding families in Fig. S2 are scattered around the sampling frame in a manner visually analogous to the sampled families in Fig. S1.

For each sampled family, we made multiple contact attempts by U.S. mail, by phone, and when possible by email, social media, or via an in-person visit. Seven of the sampled families did not respond (Table S1). These non-responders can be placed into three groups. First, two families refused to participate: one case expressed hesitancy to participate at first and subsequently hung up immediately each time we called, and one case initially had difficulty scheduling and subsequently refused to participate. Second, for three families, either the youth or primary caregiver (or both) agreed to participate but we were unable to schedule an interview with them. Finally, for two families, we had difficulty contacting them: one case seemed to have correct contact information but did not yield a response, and the other had no up-to-date contact information. We presume the respondent never received our messages.

When we could not reach a case or they declined to participate, we planned to replace that case with another similar case. Because of constraints on time and funding, we ultimately carried out this procedure for only 2 of the 7 families that were sampled but did not participate, as summarized in Table S1.

C. Differential Nonresponse. If the responding and nonresponding families differ systematically along a variable relevant to the study, differential nonresponse could produce misleading conclusions. To assess differential nonresponse, one can compare the sampled respondents (Fig. S1) with the final respondents (Fig. S2). In City A, we spoke with all 15 sampled families, so there

was no differential nonresponse. The replacement case in each of cities B and C had similar predicted and residual GPA to the originally sampled case. While these replacements do not resolve differential nonresponse along unobserved variables, they are similar on observed variables. The greatest threats of differential nonresponse come from the 5 non-responding families who were not replaced (see Table S1). Two were in City B and 3 were in City C. Of the non-replaced families, 3 fell in the middle tercile of predicted GPA and 2 fell in the upper tercile of predicted GPA within their cities. Examining the residual categories of each non-replaced family within its (city × predicted GPA) stratum, 1 had the most negative residual within its stratum, 2 were in the bottom third of non-extreme residuals within their strata, and 2 were in the middle third of non-extreme residuals within their strata. This provides some evidence that our respondents under-represents those whose GPAs were lower than their predicted values. However, the relative rarity of the non-replaced families compared to the sampled and completed families suggests that differential non-response may only have minor implications for our conclusions.

3. Interview Procedure

A team of 16 researchers conducted 114 interviews with 73 respondents. The interviews were with both young adults and their primary caregivers: 66 interviews were with 39 young adult respondents and 48 interviews were with 34 primary caregiver respondents. There were more interviews than respondents for two reasons: 1) we planned to conduct two interviews with each young adult, and 2) some interviews were interrupted and needed to be rescheduled. All interviews were conducted by a pair of researchers, and most interviews were conducted in person, although some were conducted by phone when the respondent was geographically distant or preferred to speak by phone. We conducted interviews in both English and Spanish.

Our interview guide was designed to elicit life histories from youth and retrospective accounts about the youth's life experiences from the primary caregivers, with a special focus on the time between when the young adult was 9 and 15 years old. The interview guides are included in *SI Appendix* Section 6 (Young Adult) and 7 (Primary Caregiver).

When designing the interview protocols for this study, we had to decide how much prior information about the young adult and family to make available to the interviewers. In particular, we considered whether the interviewer should know the prediction error for the young adult's GPA. On one hand, knowing the error might allow the interviewer to probe responses that might help us better uncover the factors that are leading to unexpected outcomes. On the other hand, if the interviewer probed differently based on the outcome, we risked a circular design that could be used to justify known outcomes but would not help us understand other families with unknown outcomes. Ultimately, we decided on a hybrid design that provided us the benefits of both.

Both interviewers had access to basic information about the family: dates of past interviews, the age of the youth at those interviews, and information about the youth's contact with their biological parents (e.g. whether resident or nonresident) at the last interview wave. Critically, however, the primary interviewer was unaware of the age 15 information and the GPA residual but the secondary interviewer was not. For the first part of the visit, the primary interviewer conducted the interview. At the end, the primary interviewer turned to the secondary interviewer and asked if there were any more questions. Then the secondary interviewer—who was already aware of the outcome during the entire interview—was able to probe responses that were particularly interesting given the young adult's residual and survey responses at age 15.

4. Decomposing Prediction Error: Mathematical Derivation

The proof below derives the decomposition presented in Eq (1) in the main text. When writing expectation and variance operators $E()$ and $V()$, we include subscripts on these operators to define the random variables over which expectation and variance is being taken.

$$
\begin{aligned}
&\mathrm{E}_{Y,\vec{X},S}\left(\left[Y-\hat{f}_S(\vec{X})\right]^2\right) && [1]\\
&\text{Next add 0}\\
&=\mathrm{E}_{Y,\vec{X},S}\left[\left(Y-\mathrm{E}_Y(Y\mid\vec{X})+\mathrm{E}_Y(Y\mid\vec{X})-\hat{f}_S(\vec{X})\right)^2\right] && [2]\\
&\text{Next distribute}\\
&=\mathrm{E}_{Y,\vec{X},S}\left[\left(Y-\mathrm{E}_Y(Y\mid\vec{X})\right)^2\right]+\mathrm{E}_{Y,\vec{X},S}\left[\left(\mathrm{E}_Y(Y\mid\vec{X})-\hat{f}_S(\vec{X})\right)^2\right]\\
&\qquad+2\mathrm{E}_{Y,\vec{X},S}\left[\left(Y-\mathrm{E}_Y(Y\mid\vec{X})\right)\left(\mathrm{E}_Y(Y\mid\vec{X})-\hat{f}_S(\vec{X})\right)\right] && [3]\\
&\text{Apply the law of iterated expectation to the second line}\\
&=\mathrm{E}_{Y,\vec{X},S}\left[\left(Y-\mathrm{E}_Y(Y\mid\vec{X})\right)^2\right]+\mathrm{E}_{Y,\vec{X},S}\left[\left(\mathrm{E}_Y(Y\mid\vec{X})-\hat{f}_S(\vec{X})\right)^2\right]\\
&\qquad+2\mathrm{E}_{\vec{X},S}\left(\mathrm{E}_Y\left[\left(Y-\mathrm{E}_Y(Y\mid\vec{X})\right)\left(\mathrm{E}_Y(Y\mid\vec{X})-\hat{f}_S(\vec{X})\right)\mid\vec{X},S\right]\right) && [4]\\
&\text{Next, note that in the inner expectation all terms are constant except } Y.\\
&\text{Rewrite with the inner expectation applying only to } Y.\\
&=\mathrm{E}_{Y,\vec{X},S}\left[\left(Y-\mathrm{E}_Y(Y\mid\vec{X})\right)^2\right]+\mathrm{E}_{Y,\vec{X},S}\left[\left(\mathrm{E}_Y(Y\mid\vec{X})-\hat{f}_S(\vec{X})\right)^2\right]\\
&\qquad+2\mathrm{E}_{\vec{X},S}\left[\left(\mathrm{E}_Y(Y\mid\vec{X},S)-\mathrm{E}_Y(Y\mid\vec{X})\right)\left(\mathrm{E}_Y(Y\mid\vec{X})-\hat{f}_S(\vec{X})\right)\right] && [5]\\
&\text{Because sample is random, } \mathrm{E}_Y(Y\mid\vec{X},S)=\mathrm{E}_Y(Y\mid\vec{X})\\
&=\mathrm{E}_{Y,\vec{X},S}\left[\left(Y-\mathrm{E}_Y(Y\mid\vec{X})\right)^2\right]+\mathrm{E}_{Y,\vec{X},S}\left[\left(\mathrm{E}_Y(Y\mid\vec{X})-\hat{f}_S(\vec{X})\right)^2\right]\\
&\qquad+2\mathrm{E}_{\vec{X},S}\left[\underbrace{\left(\mathrm{E}_Y(Y\mid\vec{X})-\mathrm{E}_Y(Y\mid\vec{X})\right)}_{=0}\left(\mathrm{E}_Y(Y\mid\vec{X})-\hat{f}_S(\vec{X})\right)\right] && [6]\\
&=\mathrm{E}_{Y,\vec{X},S}\left[\left(Y-\mathrm{E}_Y(Y\mid\vec{X})\right)^2\right]+\mathrm{E}_{Y,\vec{X},S}\left[\left(\mathrm{E}_Y(Y\mid\vec{X})-\hat{f}_S(\vec{X})\right)^2\right] && [7]\\
&\text{Left term is conditional variance, and does not involve } S\\
&=\mathrm{E}_{\vec{X}}\left(\mathrm{V}_Y\left[Y\mid\vec{X}\right]\right)+\mathrm{E}_{Y,\vec{X},S}\left[\left(\mathrm{E}_Y(Y\mid\vec{X})-\hat{f}_S(\vec{X})\right)^2\right] && [8]\\
&\text{Right term outer expectation is not over } Y,\\
&\text{because expectation over } Y \text{ is taken in the inner expectation}\\
&=\mathrm{E}_{\vec{X}}\left(\mathrm{V}_Y\left[Y\mid\vec{X}\right]\right)+\mathrm{E}_{\vec{X},S}\left[\left(\mathrm{E}_Y(Y\mid\vec{X})-\hat{f}_S(\vec{X})\right)^2\right] && [9]\\
&\text{Multiply right term by -1 within the square}\\
&=\underbrace{\mathrm{E}_{\vec{X}}\left(\mathrm{V}_Y\left[Y\mid\vec{X}\right]\right)}_{\text{Irreducible Error}}+\underbrace{\mathrm{E}_{\vec{X},S}\left(\left[\hat{f}_S\left(\vec{X}\right)-\mathrm{E}_Y\left(Y\mid\vec{X}\right)\right]^2\right)}_{\text{Learning Error}} && [10]
\end{aligned}
$$

An implication of this decomposition is that expected squared error would be minimized when the predicted values $\hat{f}_S(\vec{X})$ equal the conditional expectation function $\mathrm{E}_Y(Y\mid\vec{X})$, because learning error would be zero only in that case. Irreducible error takes the same value regardless of the predictions. Thus, the task of learning a prediction function that minimizes expected squared prediction error can be interpreted as equivalently seeking to estimate the conditional expectation function.

5. Supplemental Figures and Tables

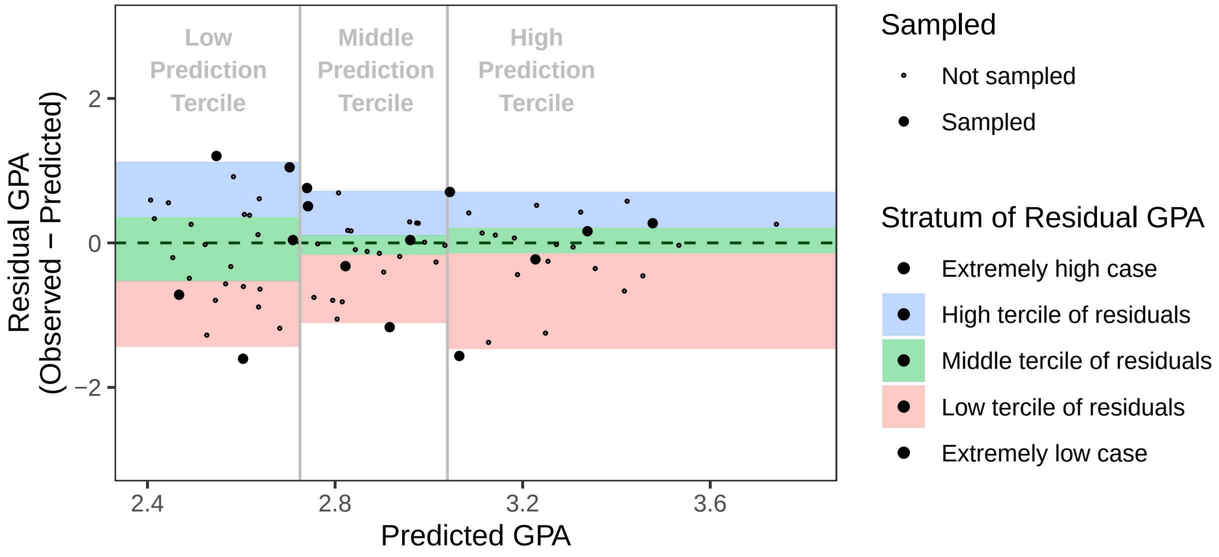

Fig. S1. Sample Selection Process. The sample was stratified by city, predicted GPA, and residual GPA. Figure depicts the sampling process for one of the three cities; the two others were analogous. There were 3 strata of predicted GPAs: low, middle, and high. There were 5 strata of residual GPAs. The two extreme strata each contained only the one family with the most extreme positive or negative residual within the city $\times$ predicted GPA stratum; these families (6 per city, 18 in total) were sampled with probability 1. The three non-extreme strata were defined by terciles of residual GPA among the remaining cases within the city $\times$ predicted GPA categories. One family was sampled at random within each stratum (9 per city, 27 in total). Overall, the stratification procedure produces a sample that contains equal numbers of families from all three cities of low, middle, and high predicted GPA, with the most extreme residual GPAs oversampled but with cases selected across the range of residual GPA.

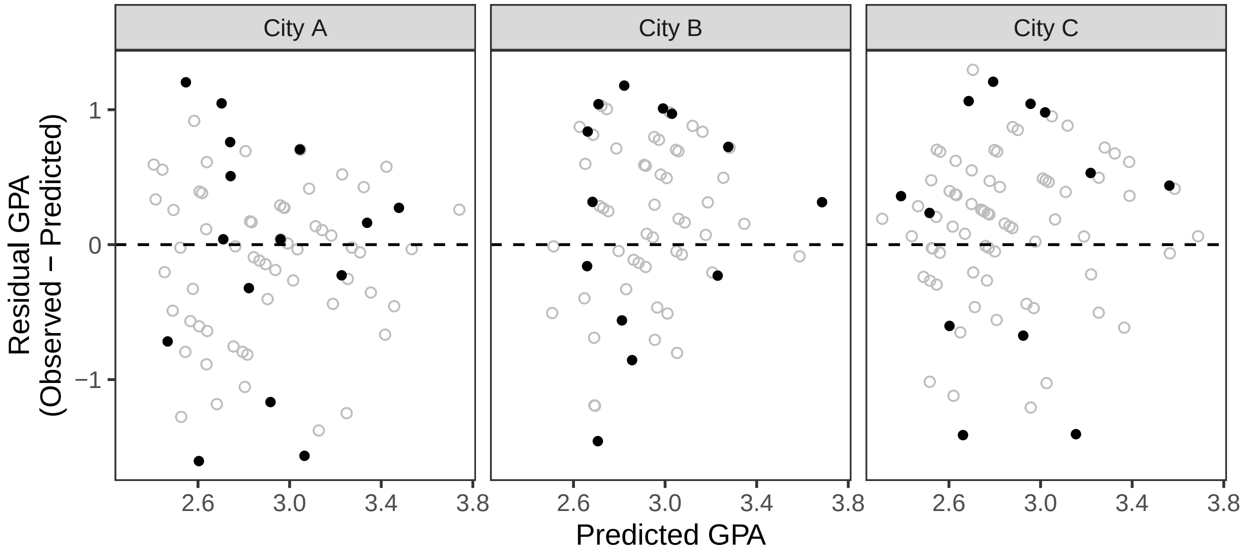

Fig. S2. Respondent Recruitment Process. Responding families (solid dots) are distributed across the range of predicted and residual GPA, with an intentional oversample at extremely large positive and negative residuals.

Table S1. Nonresponding Cases and Replacements. Seven families in the sample selection process did not respond, and two of these families were replaced with another case.

Status	Stratum	Replacement Status	Replacement Stratum
Non-contact	City C<br>High predicted GPA<br>Low residual	Not replaced	
Refusal	City B<br>High predicted GPA<br>High residual	Replaced	City B<br>High predicted GPA<br>High residual
Never scheduled	City C<br>Middle predicted GPA<br>Low residual	Not replaced	
Never scheduled	City C<br>Middle predicted GPA<br>Extremely high residual	Replaced	City C<br>Middle predicted GPA<br>High residual
Refusal	City B<br>Middle predicted GPA<br>Middle residual	Not replaced	
Non-contact	City B<br>High predicted GPA<br>Extremely low residual	Not replaced	
Never scheduled	City C<br>Middle predicted GPA<br>Middle residual	Not replaced	

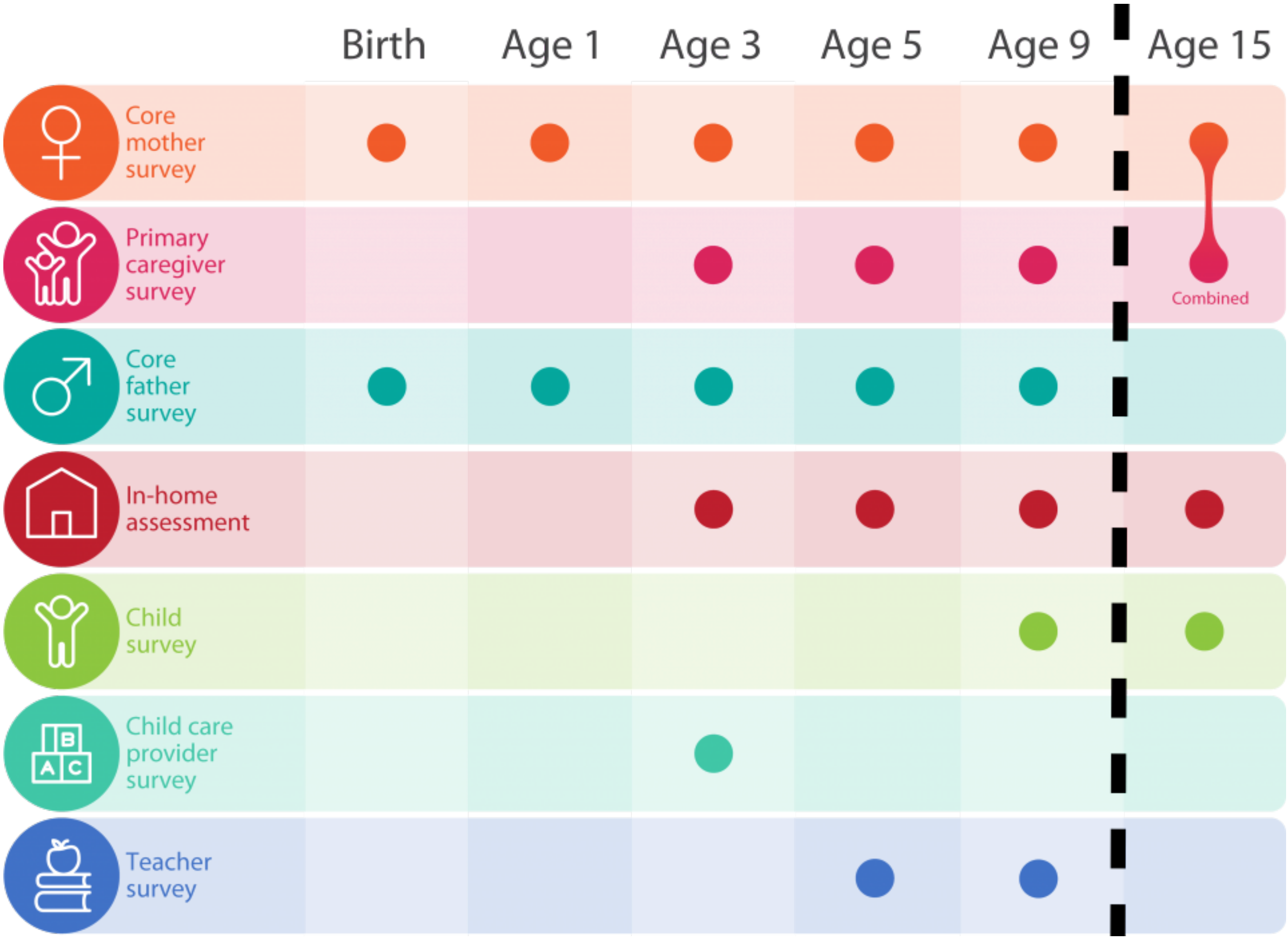

Fig. S3. Domains of predictors in the Fragile Families Challenge. Image is from the Future of Families and Child Wellbeing Study: https://ffcws.princeton.edu/.

6. Interview Guide: Young Adult Interview

Young Adult Interview Guide

Qualitative Interview Guide: Young Adult

Introduction

We want learn more about your life and what has happened since we last talked with you—when you were about 15. We want to know more about your experiences throughout your childhood and teenage years to better understand your experiences in high school and beyond.

I have some questions in mind, and I'm sure you will have some things you want to talk about too. So think of this as a conversation between friends, rather than an "interview." We will be as informal as possible. You can stop talking at any time. If I raise an issue or ask a question you don't want to talk about, just say so and we will move on to something else.

I'm going to record our conversation because this way, I can really concentrate on what you have to say. If you want me to turn off the recorder for any reason or at any time, just say so.

Please be aware that we do have a duty to inform relevant authorities if you tell us about someone harming children or your intentions to harm yourself or someone else. Everything else you tell us will be kept confidential.

We want you to choose a pseudonym, or a fake name, that we can use for your stories. We put this name on the recording and all of our files. That way your real name isn't attached to any of the information you share. Also, we will not share with your parents about anything we talk about today.

Is it okay if I turn on the recorder now? [INTERVIEWER GET VERBAL CONSENT.]

The recorder is now on. [INTERVIEWER: MAKE THIS STATEMENT AFTER YOU HAVE TURNED ON THE RECORDER. STATE YOUR NAME, THE RESPONDENT'S PSEUDONYM, THAT IT IS A YOUTH INTERVIEW, AND THE DATE.]

Any questions?

OK, let's start.

1

A. Introduction & Timeline

We're going to start off with some questions about you and your family.

A.1 Tell me a couple things about yourself that really describe *you*—who you are as a person.

A.2 Tell me about your family – what is your family like?
Probes:

- Adults?
- Siblings?
- What kind of things does your family do together?
- What kind of household rules did your parent/s have when you were coming up?
- So you've told me that (ADULTS) and (SIBLINGS) are all part of your family. Who lives in the same household with you right how? How has that changed in the last year? Tell me more about that.

We're especially interested in learning about the time period in your life kind of between the ages of 9 and 15, or between 3rd and 10th grade. A lot of our questions are going to ask you to think back to that period of time which I know can be hard. So think of this part as kind of a time machine. I'm going to just mark each point on the timeline to make sure I'm getting it right. [NOTE TO INTERVIEWER: Use a blank piece of paper.]

Let's go back to elementary school first.

A.3 Where were you living then? Tell me more about what you remember about the place and the neighborhood.

- Who was living with you then?
- How about people you didn't live with for the whole year but stayed with?

A.4 Put yourself back in time and tell me about what a typical school day was like from the time you got up in the morning and the time you went to bed at night.

- Tell me about the school you attended then. PROBE FOR SCHOOL NAME, SCHOOL CHARACTERISTICS SUCH AS EASY/HARD, LEVEL OF HOMEWORK, SCHOOL RULES, ETC). – WRITE SCHOOL ON TIMELINE
- What kind of a student were you in elementary school? Tell me more about that.
- Tell me a story about who you were in elementary school.
- What was it like during lunch in the cafeteria?
- How involved were your parents at that school? Tell me more about that.
- What extracurricular activities were you involved with at that school? Tell me more about that.
- What about activities you were involved with outside of school? Tell me more about that.
- Did parents or relatives attend games/performances/awards ceremonies?

A.5 Now let's jump ahead to middle school. Where were you living then? Tell me more about what you remember about the place and the neighborhood.

- Who was living with you then?

113

- How about people you didn't live with for the whole year but stayed with?

A.6 When you were in middle school, what was a typical school day like from the time you got up in the morning and the time you went to bed at night?

- Tell me about the school you attended then. PROBE FOR SCHOOL NAME, SCHOOL CHARACTERISTICS SUCH AS EASY/HARD, LEVEL OF HOMEWORK, SCHOOL RULES, ETC) – WRITE SCHOOL ON TIMELINE
- What kind of a student were you in middle school? Tell me more about that.
- Tell me a story about who you were in middle school.
- What was it like during lunch in the cafeteria?
- How involved were your parents at that school? Tell me more about that.
- What extracurricular activities were you involved with at that school? Tell me more about that.
- What about activities you were involved with outside of school? Tell me more about that.
- Did parents or relatives attend games/performances/awards ceremonies?

A.7 Now to high school. Where were you living then? Tell me more about what you remember about the place and the neighborhood.

- Who was living with you then?
- How about people you didn't live with for the whole year but stayed with?

A.8 When you were in high school, what was a typical school day like from the time you got up in the morning and the time you went to bed at night?

- Tell me about the school you attended then. PROBE FOR SCHOOL NAME, SCHOOL CHARACTERISTICS SUCH AS EASY/HARD, LEVEL OF HOMEWORK, SCHOOL RULES, ETC) – WRITE SCHOOL ON TIMELINE
- What kind of a student were you in X grade? Tell me more about that.
    - What grades did you get in English, Math, History, and Science?
    - Tell me about your favorite subject.
- Tell me a story about who you were in high school. How did that change over time?
- What was it like during lunch in the cafeteria?
- How involved were your parents at that school? Tell me more about that.
- What extracurricular activities were you involved with at that school? Tell me more about that.
- What about activities you were involved with outside of school? Tell me more about that.
- Did parents or relatives attend games/performances/awards ceremonies?

A.9 Okay, let's fill in these spaces on the timeline. Let's walk through each of the places you've lived since 1st grade. PROBE: Walk through each location and get the following information: how long lived there, why moved from there, why moved to the next place, who was in the household at each location.

A.10 Which of these places did you like best? Tell me a story about what that place was like.

A.11 Which of these places did you like least? Tell me a story about what that place was like.

A. 12 Let's do the same thing for schools from 1st grade on. PROBE: Walk through each school and get the following information: when they attended, why moved school to school, probing for whether the move was a natural transition, chosen because of pull factors, or due to push factors such as bullying, disciplinary reasons, or a residential move. ALSO PROBE FOR WHETHER ANY OF THESE SCHOOLS WERE ALTERNATIVE OR DISCIPLINARY SCHOOLS.

B. Education

B.1 Thinking back over your whole school career, what were the highlights? Where did you shine? Tell me a story first about middle school. How about high school?
Probe: Tell me the whole story about HIGHLIGHT from start to finish.

B.2 Thinking back over your whole school career, what were the low points? Where did you struggle? Tell me a story first about middle school. How about high school?
Probe: Tell me the whole story about LOW POINT from start to finish.

B.3 Some people say they had a teacher or other adult at school—like a counselor or a coach—that really made a difference. How about for you? Tell me a story first about middle school. How about high school?
Probe: Tell me the whole story of your connections with them.

B.4 I want to zero in on AGE AT YEAR15 when you were in X grade. Thinking back, what were you about at that time—what were you really into? What did you want to be known for? Tell me more about that.

[NOTE TO INTERVIEWER: Don't use phrase "identity project" – just fill in the blank with what the youth described above. If s/he didn't identify anything that they were known for or about, skip to B.9.]

B.5 Tell me the whole story about how you got into IDENTITY PROJECT.
Probe for timing

B.6 In terms of specific activities, what did IDENTITY PROJECT involve?

B.7 Tell me a story about what IDENTITY PROJECT meant to you then?

B.8 Who else was involved in IDENTITY PROJECT?
Were peers involved? Tell me all about them.
Were adults involved? Tell me all about them.

[NOTE TO INTERVIEWER: RE-WORD #B.9 IF YOUTH HAS NOT IDENTIFIED AN IDENTITY PROJECT.]
B.9 Some people tell us that their IDENTITY PROJECTS involved a certain way of dressing or carrying yourself. How about for you? Tell me a story about that.

B.10 What do you want to be about now? What do you want to be known for now? Tell me more about that.

B.11 Thinking back to AGE AT YEAR15, what did you NOT want to be about? How did you avoid that? Tell me a story about that.

B.12 What about now? What do you NOT want to be about? How do you avoid that? Tell me a story about that.

[*Note to interviewer: This next part about college/training school is contingent on where they're at on their journey in higher ed. Modify the questions according to their particular situation.]*

B.13 Where are you in your schooling right now? Tell me the whole story about how you got to where you are now in your schooling?
PROBE: Who has helped or hindered you along the way?

B.14 Let's zero in on teachers and counsellors at school. How specifically did they help or hinder you along the way?

B.15 Specifically, what did they tell you about trade school or college? Tell me all about those interactions. What specific steps did they encourage you to take? (PROBE FOR whether they took the ACT/SAT)

B.16 Let's zero in on your peers. How specifically did they help or hinder you along the way?

B.17 Specifically, what did they tell you about trade school or college? Tell me all about those interactions. What specific steps did they encourage you to take? (PROBE FOR whether they took the ACT/SAT)

B.18 Now let's zero in on your parents and other family members. How specifically did they help or hinder you along the way?

B.19 Specifically, what did they tell you about trade school or college? Tell me all about those interactions? What specific steps did they encourage you to take? (PROBE FOR whether they took the ACT/SAT)

B.20 Sometime people just find out things on their own about trade school or college. How about for you? Tell me more about that.

B.21 How are you thinking about schooling right now? What are your plans?
PROBE: What specific steps will it going to take to get there? Tell me how you think the process

will unfold for you?

Thinking back to high school one last time…

For those who have dropped out

B.22 Tell me the whole story—from start to finish—about how you ended up dropping out of high school.

For those who have graduated:

B.23 Sometimes it's really rough to stay in high school and graduate. Tell me the whole story about how you were able to do it?

B.24 Was there ever a point when you thought seriously about dropping out? Tell me the whole story about how you ended up staying in school.

For those in a GED program or who have a GED:

B.25 Tell me the whole story about how you ended up getting your GED.

C. Employment

C.1 Let's talk about work. Tell me about your current job/jobs. OR, IF NOT WORKING: Tell me about your last job.[1]

Probes:

- Pay, hours?
- How long working there?
- Duties/responsibilities?
- Child care arrangements (if applicable)?

C.2 Tell me the whole story about how you found this job.

C.3 Tell me about all the other jobs you've had so far.

C.4 Some people tell us that their jobs are just a way to get a paycheck. How about for you? Specifically, which of your jobs were just a way to get a paycheck? Tell me more about that.

C.5 Other people tell us that their jobs are *more* than just a way to get a paycheck. How about for you? Specifically, which of your jobs were something *more*? Tell me more about that.

[1] This includes informal jobs as well, such as working with a family member.

C.6 Tell me about how you are feeling about your job right now.

C.7 Tell me about the kind of job you'd like to have?

C.8 Think back to when you were younger, like 9 – 15 years old – what kind of job were you hoping you'd be in by the time you were in your 20s?

C.9 Some people say they have a main job, but a side hustle too. How about for you? Tell me all about it.

Probes:

- For some people a side hustle is just a way of making money. For others, it's something more. How about for you?
- When did you start this?
- Thinking back, which of these side hustles have been just a way of making money? Which have been about something more?

C.10 Of course, there's more to life than work! Some people tell us they're really involved in volunteering in their communities, or they're really into their church/mosque—something like that. Other people aren't so involved. How about for you?

Probes:

- Tell me the story about how you got involved in ACTIVITY? [Probe for when began]
- What, specifically, does being part of ACTIVITY involve?
- Who else is involved in ACTIVITY with you? Family members? Peers? Adults?
- What has being involved in X mean to you?

D. Family

Let's talk in a little more detail about your family.

D.1 I'd like to know more about PRIMARY CARE GIVER(S). [Repeat for each PCG)

Probes:

- Job – [Probe: What job did PCG have when you were in elementary school? Middle school? High school?]
- Schooling – what's the last year they completed?
- Does she/he have partner/spouse, duration of that relationship, relationship of that person with respondent? [Probe: How about when youth was in elementary school? Middle school? High school?]

D.2 How would you describe your relationship with PCG? Tell me more about that.

D.3 When you think back on your relationship with PCG, what memories stand out? (For each memory): Tell me the whole story about that from start to finish. [Probe for timing]

D.4 What's the status of your relationship with PCG right now?

D.5 (IF NOT LIVING WITH FATHER, or father not PCG) I'd like to know more about your father as well. [NOTE TO INTERVIEWER: Be sensitive in case father is deceased.]
Probes:

- Job
- Schooling – what's the last year they completed?
- Does she/he have partner/spouse, duration of that relationship, relationship of that person with respondent.

D.6 How would you describe your relationship with your father? Tell me more about that.

D.7 When you think back on your relationship with your father, what memories stand out? (For each memory): Tell me the whole story about that from start to finish. [Probe for timing]

D.8 What is the status of your relationship with your father right now?

D.9 I'd like to know about other adults who have been important in your life growing up.
Probes:

- Job
- Schooling – what's the last year they completed?
- Timing of relationship with them?

D.10 How would you describe your relationship with OTHER ADULT? Tell me more about that.

D.11 When you think back on your relationship with OTHER ADULT, what memories stand out? (For each memory): Tell me the whole story about that from start to finish. [Probe for timing]

D.12 What is the status of your relationship with OTHER ADULT right now?

IF THEY HAVE A CHILD…[IF NOT, SKIP TO D.14]

D.13 Tell me the whole story about how you became a parent.
Probes:

- Get timing
- How did you react when you first heard the news that you were going to become a mom/dad? How about the baby's OTHER PARENT? How did they react?
- What's the status of your relationship with OTHER PARENT right now? Tell me more about that.
- Some people tell us that becoming a parent really changes things in their lives. Others say it's not much of a change at all. How about for you? Tell me more about that. What about for OTHER PARENT?
- Any change in plans going for school or work?

D.14 [NOTE TO INTERVIEWER: Modify if person is with the parent of their child.] Tell me about who you are in a relationship with right now? Tell me the whole story of how you got

together with X.

Probe:

- Get timing
- How would you describe's the status of your relationship with X right now?

D.15 How about when you were in middle school and high school – tell me about any significant person that you were really talking to back then?

D.16 Tell me about your siblings. What are they like?

Probes:

- Finished high school/college?
- Working now?
- Ever get in trouble? [Timing?]
- Ever incarcerated? [Timing?]

D.17 What about any cousins you are close to? What are they like?

Probes:

- Finished high school/college?
- Working now?
- Ever get in trouble? [Timing?]
- Ever incarcerated? [Timing?]

- D.18 Some people say they have a sibling or cousin they really look up to. How about for you? Probe:Tell me a story about how you came to look up to SIBLING/COUSIN.
- Specifically, what influence has SIBLING/COUSIN had on your life?

D.19 Some people say they have a sibling or cousin that serves as a negative example. How about for you?

Probe:

- Tell me a story about how you came to see SIBLING/COUSIN as a negative example?

- Specifically, what lessons have you take from SIBLING'S/COUSIN'S life?

E. Networks/Activities

I'd like to hear about some of your friends or associates and what you all did when you hung out back when you were in middle school and high school. Let's start with middle school.

E.1 Tell me about who you looked up to when you were in middle school.

How about high school?

E.2 Now let's flip it – tell me about a person that may have been a negative example for you in middle school.

How about high school?

E.3 Tell me about who you hung out with in middle school.
In the neighborhood?
At school?

E. 4 Tell me all about what you did when you were hanging out with your friends and associates in middle school.

E. 5 Tell me about who you avoided in middle school.
Probe:
Tell me all about how you managed to avoid this person.

E.6 Back when you were in middle school, what things made you feel unsafe?
Probe:

- At home?
- In the neighborhood?
- At school?

E.7 Okay, now let's talk about hanging out in high school. Tell me about who you hung out with in high school.
In the neighborhood?
At school?

E. 8 Tell me all about what you did when you were hanging out with your friends and associates in high school.

E. 9 Tell me about who you avoided in high school.
Probe:
Tell me all about how you managed to avoid this person.

E.10 Back when you were in high school, what things made you feel unsafe?
Probe:

- At home?
- In the neighborhood?
- At school?

E. 11 Some people tell us that in middle school or high school, they really didn't hang out with anyone. They just stayed to themselves. How about for you? Tell me the whole story about how you ended up staying to yourself back then? How did this change over time?

E.12 A few people tell us that their lives are squeaky clean, but not too many. Some people smoke weed, others take a joyride with someone else's car. Some get involved in more serious

trouble. How about for you?
Probe:
Get timing (specific to middle/high school)
E.13 How about any drug use? Tell me more about that.
Probe:
Get timing (specific to middle/high school)How did this affect schoolwork?

E.14 How about your PCG? Did s/he ever have to deal with issues of using drugs or drinking too much alcohol? Tell me more about that [PROBE for affecting parenting].
Probe:
Get timing (specific to middle/high school) How did this affect schoolwork?

E.15 What about other things that can get you in trouble with the police, or even locked up? FOR EACH: Tell me more about that.
Probe:
Get timing (specific to middle/high school)

E.16 Tell me about any interactions you've had with the criminal justice system. FOR EACH: Tell me the whole story of that interaction from start to finish.
PROBE:

- Get timing (specific to middle/high school)
- Incarceration? (if so: juvenile or adult jail?)

E.17 Tell me about things you've gotten away with—things that are illegal but haven't gotten you in trouble with the police. FOR EACH: Tell me how you managed to keep these activities under the radar.
Probe:
Get timing (specific to middle/high school)

E.18 In general, what do you think of the police around here? Tell me all about how you ended up thinking about the police that way.
Probe:

- How about when you were in middle/high school?
- How about police at school?

F. Meaning

Let's flip the script and talk about something completely different. Some people your age find themselves thinking a lot about their purpose in life. Other young people are not thinking about those things at all—they're just getting on with life. How about for you? Tell me more about that.

F.1 How do you see yourself in religious or spiritual terms? *Get a term [X] from respondent.*

F.2 So if I were to ask you to describe what [X] means—If I had never heard that before—how would you describe it?

F.3 Tell me the whole story of how you came to be an X.
Probe:
Get timing (specific to middle/high school)

F.4 How big a part of your identity would you say being an X is?

F.5 Would you say that you are a "good X"? Tell me more about that?

F.6 Specifically, what does being an X involve?

F.7 On a month to month or week to week basis, what activities do you engage in as a part of being an X? *Probe for devotional activities as well as attendance at church/meetings, etc.*

F.8 How have your beliefs about religion/spiritually changed over the years? Tell me more about that.

F.9 How has your involvement in activities related to religious or spiritual things changed over the years?

F.10 How do you think being an X affected how you did in school?

G. Story of Your Life

G.1 If you were writing the story of your life right now, what would the chapters be called?[2] [May want to have a paper available for them to sketch this out]
Probe:
For each chapter: what's this chapter mainly about?

G.2 Thinking over your life story, tell me about a time that was a high point for you – where you were really happy or excited. Tell me about it in detail, as if you were writing this scene in the chapter.
Probe:
Get timing (specific to middle/high school)

G.3 Okay, now tell me about a time that was a low point for you – a moment where you felt sad or disappointed or angry about something. Tell me about it in detail, as if you were writing this scene in the chapter.
Probe:
Get timing (specific to middle/high school)

---

[2] These questions are adapted from Dan McAdams http://www.sesp.northwestern.edu/foley/instruments/interview/

G.4 Looking over your life story, can you think of a time where you changed or your situation changed such that it was a real turning point for you? Lay out that scene for me, telling me what happened and what you were feeling at the time.
Probe:
Get timing (specific to middle/high school)

G.5 Choose a time in your life where you made a wise decision – this could be something for your life or advice that you gave to someone. Tell me about it and what this moment says about you.

G.6 Thinking about the future, what will the next two chapters of your life be called?

G.7 Tell me about where you see yourself five years from now.
Probe:
What do you think you need to get there?

H. Mental Health

We're nearing the end of the interview. Before we finish up, I want to hear from you about things that can affect how we feel.

H.1 What kind of things make you feel stressed, worried, or sad right now? [3]
Probes:

- Tell me about the most recent time when you felt this way.
- How long did it last?
- How about when you were middle or high school – do you remember feeling stressed, worried, or sad? Tell me more about that.
- How did this affect your ability to do well in school?

H.2 Some people tell us they are stressed or are feeling sad after they've witnessed violence or lost people they loved. How about for you? TMMAT.
Probe:
Get timing (specific to middle/high school)

H.3 How do you get through stressful or sad times?[4]

---

[3] *We want to know about the type of acute and chronic stressors that are present in the youth's life, where they are experienced, and how they affect youths' mood. It may be helpful to probe in several domains here: School, home, jobs, community, friendships, romantic relationships, financial stress, racism/discrimination, traumatic events, health, and worry about the future or other people.*

[4] *We want to know how youth cope with the stressors that they face. Do they use problem-solving, distraction, avoidance, disengagement, seeking social support, religious coping etc. We want to know if they use the same strategies to cope with different stressors, and whether they use different*

Probes:

- Ever been on medicine for depression?
- Ever go to the hospital for depression?
- Get timing (specific to middle/high school)

H.4 Some people say they struggle with anger from time to time. How about for you?
Probes:

- What kind of things make you angry?
- How do you cope when you feel angry?
- How about when you were middle or high school – do you remember struggling with anger? Tell me more about that.
- How did this affect your ability to do well in school?

H.5 Tell me a story about the last time you felt really angry. Tell me the whole story from start to finish.

H.6 Some people tell us they've gotten so angry that they broke something or tried to hurt someone. How about for you? Tell me the whole story about the last time that happened?
Probes:

- Ever been on medicine for anger?
- Ever go to the hospital for anger?

Get timing (specific to middle/high school)
H.7 Some people say that other people—family, friends, ministers, health professionals—have really helped with getting through these tough times. Other people say they struggle alone. How about for you? TMMAT. [5]

H.8 Thinking back over your life story, what moments brought you joy?

I. Conclusion

We really appreciate the time that you've spent with us today sharing about yourself.

I.1 At this point in your life, what advice would you have for your younger self, like when you were in middle school?

I.2 What's it like being a young adult like yourself in this neighborhood?

---

strategies for the same stressor when they are in different settings. We want to know whether some coping strategies work in one setting/community, but not in another.

[5] *We want to get at the type of support the youth receives – financial, emotional, or other instrumental support. Also, how do these support networks help? Sometimes friends and family members can be supportive and just lend an open ear, while sometimes they can actually give advice or critical suggestions.*

How about in this city?
How about in America?

I.3 Is there anything else you'd like to tell me before we move on? Anything that you think I've missed or that I should know?

Thank you so much. We have a few questions we'd like to ask related to the survey you took before. As we said before, you may be nominated for us to come for a second interview, so perhaps we'll be able to talk again.

Material Hardship Supplement **[If returning for a second interview]**

[Modify for housing/living situation if living independently]

1. It can be really hard to make ends meet. Over the last 12 months, tell me about a time when you had to pay just part of a bill or had to put off paying a bill until the next month.
Probes:
How often does that happen?
How do you decide what gets paid and what doesn't?

2. Tell me about your housing. What are you paying right now in rent? How has that changed over the last year?
Probes:
Do you get any help from the government for rent?
How about utilities?

3.Who else shares the responsibility for the rent and/or other household expenses? Tell me all about that arrangement. How has that changed over the last year?

4. Some people say "there's a lot of month left at the end of the money." How about for you? When does the money run out?

5. Generally, what are your coping strategies? Tell me all about that.

- Probe: any sources of unreported income.

6. Let's start with last month. What was that like?
Probe:
How did those months compare with the rest of last year?

7. Overall, how has the past year been for you financially? Tell me more about that.
Probe:
How does this year compare to last year? And the year before that?

127

7. Interview Guide: Parent Interview

Primary Caregiver Interview Guide

Qualitative Interview Guide:

Primary Caregiver

Introduction

We want to learn more about [FOCAL CHILD's] life and what has happened since we last talked with you around [FC'] 15th birthday. We want to know more about your and your child's experiences throughout their childhood and teenage years to better understand their experiences in high school and beyond.

I have some questions in mind, and I'm sure you will have some things you want to talk about too. So think of this as a conversation between friends, rather than an "interview." We will be as informal as possible. You can stop talking at any time. If I raise an issue or ask a question you don't want to talk about, just say so and we will move on to something else.

Please be aware that we do have a duty to inform relevant authorities if you tell us about someone harming children or your intentions to harm yourself or someone else. Everything else you tell us will be kept confidential.

I'm going to record our conversation because this way, I can really concentrate on what you have to say. If you want me to turn off the recorder for any reason or at any time, just say so.

We want you to choose a pseudonym, or a fake name, that we can use for your stories. We put this name on the recording and all of your files. That way your real name isn't attached to any of this information you share. Also, we will not talk to [ADOLESCENT] about what we talk about today.

Is it okay if I turn on the recorder now? [INTERVIEWER GET VERBAL CONSENT.]

The recorder is now on. [INTERVIEWER: MAKE THIS STATEMENT AFTER YOU HAVE TURNED ON THE RECORDER. STATE YOUR NAME, THE RESPONDENT'S PSEUDONYM, THAT IT IS A YOUTH INTERVIEW, AND THE DATE.]

Any questions?

OK, let's start.

1

A. Introduction

A.1 Tell me a little about your children [PAUSE for open ended response. PROBE for names and ages_

NOTE TO INTERVIEWER: Insert young adult's name for FOCAL CHILD AND FC]

A.2 We're just going to be focusing on FOCAL CHILD (FC) today. Tell me about her/him.

We're especially interested in learning about the time period in FC's life kind of between the ages of 9 and 15, or between 3rd and 10th grade. A lot of our questions are going to ask you to think back to that period of time. So think of this part as kind of a time machine. I'm going to just mark each point on the timeline to make sure I'm getting it right. [NOTE TO INTERVIEWER: Use a piece of paper for this.]

Let's go back to when FC was in elementary school first.

A.3 Where were you living then? Tell me more about what you remember about the place and the neighborhood.

- Who was living with you then?
- How about people you didn't live with for the whole year but stayed with?

A.4 Tell me about the school that FC went to then. PROBE FOR SCHOOL NAME, SCHOOL CHARACTERISTICS SUCH AS EASY/HARD, LEVEL OF HOMEWORK, SCHOOL RULES, ETC). – WRITE SCHOOL ON TIMELINE

- What kind of student was FC in elementary school?
- How were you involved at that school? Tell me more about that.
- What extracurricular activities was FC involved with at that school? Tell me more about that.
- What about activities FC was involved with outside of school? Tell me more about that.

A.5 Now let's jump ahead to when FC was in middle school. Where were you living then? Tell me more about what you remember about the place and the neighborhood.

- Who was living with you then?
- How about people you didn't live with for the whole year but stayed with?

A.6 Tell me about FC's middle school. PROBE FOR SCHOOL NAME, SCHOOL CHARACTERISTICS SUCH AS EASY/HARD, LEVEL OF HOMEWORK, SCHOOL RULES, ETC) – WRITE SCHOOL ON TIMELINE

- What kind of student was FC in middle school?
- How were you involved at that school? Tell me more about that.
- What extracurricular activities was FC involved with at that school? Tell me more about that.
- What about activities FC was involved with outside of school? Tell me more about that.

A.7 Now to when FC was in high school. Where were you living then? Tell me more about what you remember about the place and the neighborhood.

- Who was living with you then?
- How about people you didn't live with for the whole year but stayed with?

A.8 Tell me about FC's high school. PROBE FOR SCHOOL NAME, SCHOOL CHARACTERISTICS SUCH AS EASY/HARD, LEVEL OF HOMEWORK, SCHOOL RULES, ETC) – WRITE SCHOOL ON TIMELINE

- What kind of student was FC in high school?
- How were you involved at that school? Tell me more about that.
- What extracurricular activities was FC involved with at that school? Tell me more about that.
- What about activities FC was involved with outside of school? Tell me more about that.

A.9 Okay, let's fill in these spaces on the timeline. Let's walk through each of the places you've lived starting when FC was in 1st grade.

PROBE: Walk through each location and get the following information: how long lived there, why moved from there, why moved to the next place, who was in the household at each location.

[NOTE TO INTERVIEWER: SKIP A.10 – A.12 IF THEY ALREADY DISCUSSED THIS FOR THEIR MOVES.]

A. 10 Thinking back to all the places you've lived throughout FC's childhood, which of these places did you like best? Tell me a story about what that place was like. Which do you think FC liked best? TMMAT.

A.11 Which of these places did you like least? Tell me a story about what that place was like. Which do you think FC liked least? TMMAT.

B. Education

Let's go back to talking a little more about FC's school years.

B.1 We're going to use this timeline and walk through each of the schools FC went to starting from 1st grade.

PROBE: Walk through each school and get the following information: when they attended, why moved school to school, probing for whether the move was a natural transition, chosen because of pull factors, or due to push factors such as bullying, disciplinary reasons, or a residential move. ALSO PROBE FOR WHETHER ANY OF THESE SCHOOLS WERE ALTERNATIVE OR DISCIPLINARY SCHOOLS.

B.2 I'm particularly interested in how FC ended up in FIRST HIGH SCHOOL. Tell me the whole story from start to finish. Tell me everything about that school, and what going there was like

3

131

for you. (REPEAT FOR EACH HIGH SCHOOL).

B.3 Thinking back over your FCs school career, what were the highlights? Where did she/he shine? Tell me first a story from middle school. How about high school?

B. 4 Thinking back over FC's whole school career, what were the low points? Where did she/he struggle? Tell me first a story from middle school. How about high school?

B.5 Some parents tell us that their child had teacher or other adult at school—like a counselor or a coach—that really made a difference. How about for FC? Tell me the whole story of FCs relationships with TEACHER/OTHER ADULT AT SCHOOL.
Probe:
Get timing (middle school/high school?)

For those with FCs who have dropped out

B.6 Tell me the whole story—from start to finish—about how FC ended up dropping out of high school.

For those with FCs who have graduated:

B.7 Sometimes it's really rough to stay in high school and graduate. Tell me the whole story about how FC was able to do it?

B.8 Was there ever a point when FC thought seriously about dropping out? Tell me the whole story about how FC ended up staying in school.

For those with FCs in a GED program or who have a GED:

B.9 Tell me the whole story about how FC ended up enrolling in GED classes/getting her/his GED.

B.10 How is FC thinking about schooling right now? What are FC's plans?
PROBE: What specific steps do you think will it going to take for FC to get there? Tell me how you think the process will unfold for FC?

C. Parenting

Supporting your child throughout school is just one part of being a parent. I want to hear your thoughts about parenting more generally.

C.1 When FC was growing up, describe *you* as a parent.

Probe:
Get timing (middle school/high school)

C.2 What parenting strategies did you try? How did your strategies compare with your parents' parenting strategies? The strategies of other parents with kids your kids' age? How well did those work with FC? TMMAT.
Probe:
How did these strategies change from middle school into high school?

C.3 How was parenting FC different from parenting your other children? TMMAT.

C.4 I'm interested in what was it like to be the parent of FC when FC was in middle school. Tell me a story about that. How about high school?

C.5 What were your successes parenting FC, and where do you feel you fell short?

D. Family & Friends

Let's talk for a little bit about FC's family and friends.

D.1 How well does FC get along with her/his siblings? Tell me a story about that.

D.2 Tell me about other people who have been important in FC's life. Let's start with other family members who are important in your family's life.
PROBE:
Get timing (middle school/high school) specifically for how they relate to FC

D. 3 How about others who have been important in your family's life?
PROBE:
Get timing (middle school/high school) specifically for how they relate to FC

D.4 Tell me more about your other children. What are they like?
Probes: Get timing for these events relative to FC in middle school/high school

- Finished high school/college?
- Working now?
- Ever get in trouble?
- Ever incarcerated?

D.5 What about cousins FC is close to? What are they like?
Probes: Get timing for these events relative to FC in middle school/high school

- Finished high school/college?
- Working now?
- Ever get in trouble?
- Ever incarcerated?

133

D.6 Some parents tell us their child has a a sibling or cousin they really look up to. How about for FC? What influence do you think SIBLING/COUSIN has had on your child's life?

D.7 Some parents tell us their child has a sibling or cousin that serves as a negative example. How about for FC? What influence do you think SIBLING/COUSIN has had on your child's life?

D.8 A few parents tell us that their kids' lives are squeaky clean, but not too many. Some have kid who've smoked weed, others have taken a joyride with someone else's car, things like that. Some have gotten involved in more serious trouble. How about for FC?
Probe:
Get timing (specific to middle/high school)

D.9 How about any substance use? Tell me more about that.
Probe:

- Get timing (specific to middle/high school)
- How do you think this affected FC's schoolwork?

D.10 How about for you? Did you ever even struggle with substances? Tell me more about that. [PROBE for affecting parenting].
Probe:

- Get timing (specific to middle/high school)
- How do you think this affected FC's schoolwork?
- Did FC's other parent deal with the same stuff? TMMAT.

D.11 What about other things that can get a child in trouble with the police, or even locked up? FOR EACH: Tell me more about that.
Probe:
Get timing (specific to middle/high school)

D.12 Tell me about any interactions FC has had had with the criminal justice system. FOR EACH: Tell me the whole story of that interaction from start to finish.
Probe:

- Get timing (specific to middle/high school)
- Incarceration? (if so: juvenile or adult jail?)

D.13 Tell me about things FC got away with—things that are illegal didn't get them in trouble with the police.
Probe:
Get timing (specific to middle/high school)

F. Story of Your Life as a Parent

134

F.1 If you were writing the story of your life as a parent right now, what would the chapters be called?[1] [May want to have a paper available for them to sketch this out]
Probe:
For each chapter: what's this chapter mainly about?

F.2 Thinking over your life as a parent, tell me about a time that was a high point for you – where you were really happy or excited. Tell me about it in detail, as if you were writing this scene in the chapter.
Probe:
Get timing (specific to middle/high school)

F.3 Okay, now tell me about a time that was a low point for you as a parent – a moment where you felt sad or disappointed or angry about something. Tell me about it in detail, as if you were writing this scene in the chapter.
Probe:
Get timing (specific to middle/high school)

F.6 Looking over your life as a parent, can you think of a time where you changed or your situation changed such that it was a real turning point for you? Lay out that scene for me, telling me what happened and what you were feeling at the time.
Probe:
Get timing (specific to middle/high school)

F.7Thinking about your future as a parent, what will the next two chapters of your life be called?

F.8 Tell me about where you see FC five years from now.

F.9 What do you think you need to do to help her/him get there?

G. Mental Health

One of the things that can affect how we parent is how we feel.

G.1 What kind of things have made you feel stressed, worried, or sad? [2]

---

[1] These questions are adapted from Dan McAdams
http://www.sesp.northwestern.edu/foley/instruments/interview/

[2] *We want to know about the type of acute and chronic stressors that are present in the youth's life, where they are experienced, and how they affect youths' mood. It may be helpful to probe in several domains here: School, home, jobs, community, friendships, romantic relationships, financial stress, racism/discrimination, traumatic events, health, and worry about the future or other people.*

Probes:

- Tell me about the most recent time when you felt this way.
- How long did it last?
- How about when FC was in middle school? In high school? Tell me more about that.

G.2 Some people tell us they are stressed or are feeling sad after they've witnessed violence or lost people they loved. How about for you? TMMAT.
Probe:
Get timing (specific to middle/high school)

G.3 How do you get through stressful or sad times?[3]
Probes:

- Ever been on medicine for depression?
- Ever go to the hospital for depression?
- Get timing (specific to middle/high school)

G.4 Some people say they struggle with anger from time to time. How about for you?
Probes:

- What kind of things make you angry?
- How do you cope when you feel angry?
- Ever been on medicine for anger? (Timing)
- How about when you FC was in middle or high school – do you remember struggling with anger? Tell me more about that.

G.5 Thinking back over this long time of parenting FC, what moments brought you joy?

H. Employment & Budgets

We're getting near the end of the interview, and we're going to switch gears and talk about work and making ends meet.

H.1 Tell me about work. What's your job situation like now?
Probe:

- About how much do you get paid each month?
- How long have you been there?
- How long do you want to stay?
- Benefits?

---

[3] *We want to know how youth cope with the stressors that they face. Do they use problem-solving, distraction, avoidance, disengagement, seeking social support, religious coping etc. We want to know if they use the same strategies to cope with different stressors, and whether they use different strategies for the same stressor when they are in different settings. We want to know whether some coping strategies work in one setting/community, but not in another.*

H.2 Tell me about (other) sources of income.
Probe: income from second jobs, informal jobs, overtime pay, and government benefits like SNAP, WIC, TANF, disability

H.3 Where were you working when FC was:
in elementary school?
Probe: full-time/part-time, how long
in middle school?
Probe: full-time/part-time, how long
in high school?
Probe: full-time/part-time, how long

It can be really hard to make ends meet. I'm going to ask you to try to think back in time to when FC was younger about what it was like to hold things together financially.

H.4 Let's think about food first, which can get super expensive when you have to feed kids who are growing more and more every day. When FC was in middle school, was there a time you remember where you got free food or meals from some place in order to add on to what you could afford? TMMAT.
Probe:
How about in high school?

H.5 Sometimes parents skip meals even when they're hungry because there's not enough money for food. Did that happen for you when FC was in middle school? TMMAT.
Probe:
How about in high school?

H.6 Let's talk about how much it costs to keep your house running. Thinking back to when FC was in middle school, was there a time where you couldn't pay the full amount for rent or mortgage? TMMAT.
Probe:
How about in high school?

H.7 Have you ever had a situation where you had to leave a place because you couldn't pay the rent? TMMAT
Probe:
Get timing (middle school/high school for FC?)

H.8 Sometimes when money gets tight people tell us that they move in or stay with other people for a while. How about for you? TMMAT.
Probe:
Get timing (middle school/high school for FC?)

H.9 We've heard from some people that sometimes it wasn't an option for them to stay with others when they didn't have housing, so they stayed in a shelter or a car for a while. How about for you? TMMAT.
Probe:
Get timing (middle school/high school for FC?)

H.10 How about those utility bills – was there a time when FC was in middle school where you had to not pay a gas, water, oil, or electric bill? TMMAT.
Probe:
- How about in high school?
- Did the utility company ever shut off service? TMMAT (probe for timing)

H.11 How about phone service – whether it's landline or cellphone – did you ever get that cancelled because you didn't have enough money for the bill when FC was in middle school?
Probe:
How about in high school?

H.12 Sometimes friends and family pitch in for us when money is tight. Thinking back to when FC was in middle school, did that ever happen for you? TMMAT.
Probe:
- How about in high school?
- Were you ever called on by friends and family to help them out with bills? TMMAT.

H.13 Last question where I'll ask you to go back in time! We all know that health care costs are getting crazier. Thinking back to when FC was in middle school, was there a time when someone living with you needed to see a doctor or go to the hospital, but couldn't because it was too expensive? TMMAT.
Probe:
How about in high school?

H.13 Some people say "there's a lot of month left at the end of the money." How about for you? When does the money run out?

H.14 Overall, how has the past year been for you financially? Tell me more about that.
Probe:
How does this year compare to last year?

I. Conclusion

I.1 You've been so generous with your time. I really appreciate it. We're at the end, and I just want to ask you to tell me about where you see yourself and your family five years from now.

I.2 Is there anything else you'd like to tell me before we end? Anything that you think I've missed or that I should know?

References

1. MJ Salganik, et al., Measuring the predictability of life outcomes with a scientific mass collaboration. *Proc. Natl. Acad. Sci.* **117**, 8398–8403 (2020).
